# Development of a novel rDNA based plasmid for enhanced cell surface display on *Yarrowia lipolytica*

**DOI:** 10.1186/2191-0855-2-27

**Published:** 2012-05-20

**Authors:** Siyavuya Ishmael Bulani, Lucy Moleleki, Jacobus Albertyn, Ntsane Moleleki

**Affiliations:** 1 Council for Scientific and Industrial Research, CSIR Bioscences, P.O. Box 395, Pretoria, 0001, South Africa; 2 Department of Microbiology and Plant Pathology, University of Pretoria, Pretoria, 0001, South Africa; 3 Department of Microbial, Biochemical and Food Biotechnology, University of the Free State, P.O. Box 339, Bloemfontein, 9300, South Africa

**Keywords:** mCherry, rDNA vector, YlCWP1, *Yarrowia lipolytica*, Cell surface display

## Abstract

In this study, a novel rDNA based plasmid was developed for display of heterologous proteins on the cell surface of *Yarrowia lipolytica* using the C-terminal end of the glycosylphosphatidylinositol (GPI) anchored *Y. lipolytica* cell wall protein 1 (YlCWP1). mCherry was used as a model protein to assess the efficiency of the constructed plasmid. *Y. lipolytica* transformants harbouring the expression cassettes showed a purple colour phenotype on selective YNB-casamino plates as compared to control cells indicating that mCherry was displayed on the cells. Expression of mCherry on cells of *Y. lipolytica* was confirmed by both fluorescent microscopy and flow cytometry. Furthermore, SDS-PAGE analysis and matrix-assisted laser desorption/ionization (MALDI)-time-of (TOF)-mass spectrometry (MS) peptide mass fingerprinting (PMF) confirmed that the protein cleaved from the yeast cells using enterokinase was mCherry. Efficient cleavage of mCherry reported in this work offers an alternative purification method for displayed heterologous proteins on *Y. lipolytica* cells using the plasmid constructed in this study. The developed displaying system offers great potential for industrial production and purification of heterologous proteins at low cost.

## Introduction

Since the first development of a cell surface display system on bacteriophage by ([Smith 1985]), various yeast cell surface displaying systems have been developed for expression of heterologous proteins ([Sergeeva et al. 2006]). Yeast cell surface display has been used as a method of choice for expression of heterologous proteins. This is because yeast cell surface display is convenient, shows ease of handling of displayed heterologous proteins and has been found to be comparatively stable against environmental changes ([Inaba et al. 2010]). In addition, the advantage of yeast cell surface display over bacterial display is that yeast has a post-translational modification system that resembles the mammalian system for efficient processing and folding of proteins ([Kondo and Ueda,2004]). Cell surface displaying systems in yeast, in particular *Saccharomyces cerevisiae*, have been studied extensively ([Kondo and Ueda, 2004];[ Furukawa et al. 2006]). Although *S. cerevisiae* emerged as the most favourable microorganism for displaying heterologous proteins ([Kondo and Ueda, 2004]), hyperglycosylation of expressed proteins has remained a major drawback ([Gemmill and Trimble, 1999]). Hyperglycosylation of heterologous proteins has a potential to affect protein activity ([Wang et al. 2007]). Other yeasts, such as *Pichia pastoris* have been reported to express heterologous proteins with reduced glycosylation ([Choi et al. 2003]). More recently, cell surface display of active heterologous proteins in both *Y. lipolytica* and *P. pastoris* has gained momentum.

Amongst the non-conventional yeasts, *Y. lipolytica* remains one of the most attractive hosts for heterologous protein production [(Muller et al. 1998]). A series of molecular tools for heterologous protein expression in *Y. lipolytica* have been developed [(Nicaud et al. 2002]; [Madzak et al. 2004]). A surface displaying vector for immobilization of proteins on *Y. lipolytica* has been constructed by [Yue et al. (2007]). The vector carries zeta elements (LTRs from Ylt1 retrotransposon), which allows it to integrate either by homology in *Y. lipolytica* strains carrying Ylt1, or by non-homologous integration into Ylt1-free strains [(Nicaud et al. 1998]; [Pignede et al. 2000a]; [Juretzek et al. 2001]). The zeta based plasmid employs the C-terminal end of the *YlCWP1* for cell surface display of proteins. A wide range of heterologous proteins have been successfully displayed on *Y. lipolytica* cell surface using the zeta-based displaying plasmid [(Ni et al. 2009]; [Liu et al. 2009,^2010^]; [Yu et al. 2010]). More recently, studies by ([Yuzbasheva et al. 2011]) reported five genes encoding YlCWP. All identified proteins were used successfully to display active Lip2 lipase on *Y. lipolytica* employing a zeta-based plasmid *.* The displaying plasmid of ([Yue et al. 2007]) has been used to carry *FLO1* for cell surface display of an active mannanase on *Y. lipolytica* cells ([Yang et al. 2009]).

As an alternative approach, in this study a new vector for cell surface display on *Y. lipolytica* cells was developed. The construction of the displaying vector is based on the rDNA autocloning pKOV410. The vector integrates homologously into the yeast ribosomal cluster. The new plasmid employs the growth-phase dependent promoter, hp4d, for heterologous expression of proteins ([Madzak et al. 2004]), the pre-pro Lip2 secretion signal ([Pignede et al. 2000a,b]) for directing secretion, the *ura3d4* defective marker for multicopy integration [(Le Dall et al. 1994]) and the Lip2 terminator ([Barth and Gaillardin, 1996]). In this study, we successfully constructed a novel rDNA based plasmid for enhanced cell surface display of heterologous proteins on *Y. lipolytica* using YlCWP1 as a membrane anchor. The ability of the new rDNA based plasmid to display heterologous proteins on *Y. lipolytica* cell surface was tested using the fluorescent protein mCherry as a reporter. We observed that yeast transformants displaying mCherry showed colour change on YNB-casamino selective plates and culturing medium. The displayed mCherry was confirmed by fluorescence microscopy and flow cytometry. In addition, mCherry was easily cleaved from the yeast cells and detected using SDS-PAGE.

## Material and methods

### Strains and media

*Escherichia coli* XL10 Gold cells (Stratagene) were used for cloning and plasmid propagation. *E. coli* transformants were grown in 5.0 ml Luria-Bertani (LB) broth or agar plates at 37°C overnight ([Sambrook et al. 1989]). When necessary, 30 μg/ml of kanamycin or 50 μg/ml of ampicillin was added. *Yarrowia lipolytica* Po1f strain ( *MatA, leu2-270, ura3-302, xpr2-322, axp1-2*; [Madzak et al. 2000]) was used as host for cell surface display. In addition, *Y. lipolytica* Po1f strain genomic DNA was used for amplification of the C-terminal end of the gene encoding glycosylphosphatidylinositol anchored cell wall protein ( *GPI-CWP1*). Yeast transformants were selected on YNB- casamino acid plates (0.17% YNB without amino acids and ammonium sulfate, 1% glucose, 0.1% casamino acids, 0.1% sodium glutamate and 1.5% agar). For expression of immobilised proteins on *Y. lipolytica* cell wall, the yeast was grown in 100 ml YPD (1% yeast extract, 2% bacto-peptone and 2% glucose). For solid media, 1.5% agar was added.

### Plasmids

**Figure 1:**
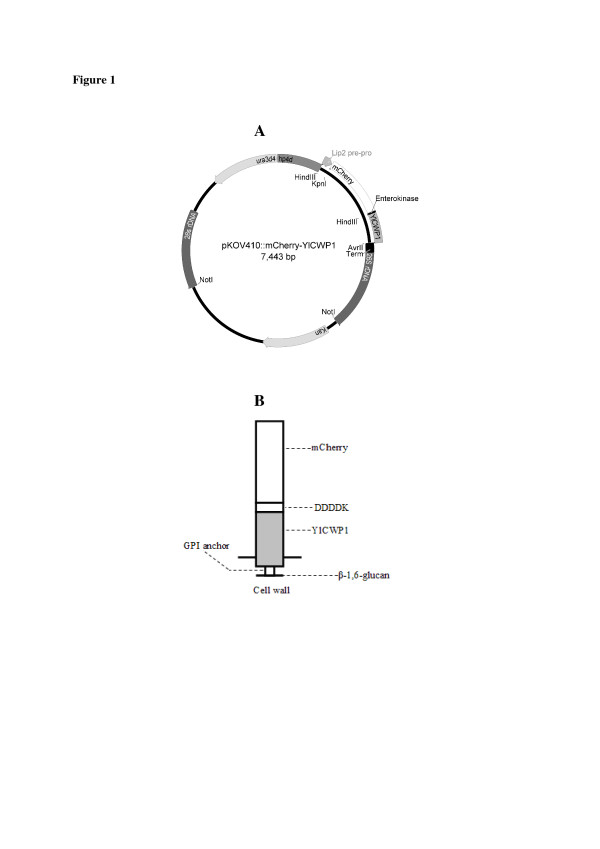
**Schematic plasmid map of pKOV410*-mCherry-YlCWP1*expression vector and of the cell wall fusion proteins.** ( **A**) *mCherry-YlCWP1* fusion gene in plasmid pKOV410- *mCherry-YlCWP1* under the transcriptional control of hp4d promoter, pre-pro Lip2 for secretion of fusion proteins and terminator (Term). The plasmid contains *URA3d4* and *KanR* markers for selection. ( **B**) mCherry-YlCWP1 covalently binds to β-1,6-glucan in the cell wall of *Y. lipolytica* through glycosylphosphatidylinositol (GPI) anchor. The plasmid map was constructed using Geneious v5.5 ([Drummond et al. 2011]).

The plasmid pKOV410 (Figure 1), an rDNA based multicopy vector was constructed at the Department of Microbial, Biochemical and Food Biotechnology, University of Free State, South Africa. Plasmid pRSET-B harbouring the gene encoding mCherry was kindly supplied by Dr. Lucy Moleleki of the Department of Microbiology and Plant Pathology, University of Pretoria, South Africa. All sub-clonings of PCR products were done using pGEM®-T Easy vector (Promega, Madison, USA).

### PCR amplification

Thermocycling reactions were carried out using MJ Mini Personal Thermal Cycler (BIO-RAD). PCR amplifications were performed using *Taq* polymerase (Fermentas). A PCR reaction mixture was prepared containing 1/10 volume reaction buffer with magnesium chloride, 10 mM dNTPs, 1.0 μM each of primer, 0.625 U *Taq* polymerase, 5 μg of DNA and topped up to a total volume of 50 μl with distilled water. The thermal cycling conditions included an initial denaturation at 98°C for 30 sec, followed by 30 cycles of denaturation at 98°C for 10 sec, annealing at 55°C for 20 sec and extension at 72°C for 1 min, with a final extension step of 75°C for 5 min and held at 4°C.

### DNA extraction, purification, restriction digestion and transformation

Yeast total genomic DNA from *Y. lipolytica* was extracted as described by ([Sambrook et al. 1989]). Plasmids from *E. coli* transformants were isolated using Plasmid Isolation Kit (BioFlux) according to the manufacturer’s instructions. PCR products were gel purified using Gel Extraction Kit (BioFlux) according to the manufacturer’s instructions. Bacterial transformants were plated out onto LB agar plates containing 30 μg/ml of kanamycin or 50 μg/ml of ampicillin. *Yarrowia lipolytica* transformation was performed as described by ([Xuan et al. 1988]).

### Construction of recombinant vector for surface display of mCherry

Primers for amplification of *YlCWP1* C-terminal end were designed based on the sequence of the gene (GeneBank Accession number: **AY084077**). The *YlCWP1* gene was amplified from the genomic DNA of *Y. lipolytica* Po1f strain using the forward primer Cwp1_F (5’- GGTACCATT **AAGCTT**ATGGGCAACGGTTACGCCGT-3’; underlined and bold bases indicate a *Kpn*I and *Hind*III sites respectively) and the reverse primer Cwp1_R (5’-GGG CCTAGGCAATTAAGCTGTAATGAGGAG-3’; underlined bases indicate an *Avr*II site). PCR amplification was done as described above. PCR products (369 bp) were separated by agarose gel electrophoresis and recovered using a Gel Extraction Kit (BioFlux). Purified PCR products were subcloned into pGEM®-T Easy vector (Promega, USA) following the manufacturer’s instructions and transformed into *E. coli* XL10 Gold. Recombinant vectors harbouring PCR products were extracted from *E. coli* transformants and purified using a Plasmid Isolation Kit (BioFlux).

To construct a recombinant cell surface displaying vector containing *mcherry*, the primers for amplification of the gene encoding mCherry were designed according to the sequence of the gene (GeneBank Accession number: **HM771696**) and published work by ([Lagendijk et al. 2010]) with primers mC-F (5’-AAAGGTACCGGAATGGTGAGCAAGGGCGAG-3’; underlined bases indicate a *Kpn*I site) and mC-R: (5’-TTT AAGCTTTAC **CTTGTCGTCGTCGTC***ATCGAT*TTTCTTGTACAGCTCGTCCAT-3'; underlined bases indicate a *Hind*III site, bold bases encode enterokinase cleavage sequence and italic bases indicate a *Cla*I site). PCR amplification was performed as described above. The plasmid pRSET-B was used as a template for amplification of *mcherry*. PCR products (756 bp) were digested with *Kpn*I and *Hind*III and ligated into pGem- *YlCWP1* digested with the same enzymes. The ligation reaction mixture was transformed into *E. coli* XL10 Gold. Recombinant vectors harbouring fusion genes were extracted from *E. coli* transformants and purified as described above. The resulting plasmid harbouring *mcherry* was named pGem- *cherry-YlCWP1*. The plasmid was subsequently digested with *Kpn*I and *Avr*II and digests ligated into pKOV410 digested with the same enzymes and transformed into *E. coli* XL10 Gold. Generated plasmid harbouring the fusion gene *mcherryYlCWP1* was named pKOV410- *mcherryYlCWP1* (Figure 1).

### Yeast transformation

Recombinant plasmids pKOV410-*mcherry-YlCWP1* and pKOV410 *-YlCWP1* were digested with *Not*I and the expression cassettes separated by agarose gel electrophoresis. Bands of interest were recovered using a Gel Extraction Kit (BioFlux) and transformed into *Y. lipolytica* Po1f by lithium acetate method ([Xuan et al. 1988]). Transformants were selected on YNB-casamino plates and isolated after 1 to 3 weeks of incubation at 28°C. Genomic DNA was extracted using the method of ([Chen et al. 1997]) and used as a template to confirm integration of the expression cassettes into the yeast genome as described previously. The primer pair mC-F and Cwp1_R was used to amplify *mcherry-YlCWP1* fusion gene (data not shown) to check the integration of the fusion gene in yeast genome. The yeast transformants carrying pKOV410- *mcherry-YlCWP1* expression cassettes were denoted as Yl-mch1 and those carrying pKOV410 *-YlCWP1* were denoted as Yl-p410.

### Culture conditions

Yeast transformants, Yl-mch1 and Yl-p410 were inoculated into 25 ml of YPD medium and incubated overnight at 28°C with shaking at 200 rpm. When the culture reached an optical density at 600 nm (OD_600_) of 2–3, cells were re-suspended to an OD_600_ of 1.0 in 100 ml YPD and incubated at 28°C with shaking at 200 rpm from 96 hours. Cultures were grown in shake flasks under aerobic conditions.

### Analysis using fluorescence microscopy and flow cytometry

For detection of mCherry displayed on the cell wall of *Y. lipolytica* using YlCWP1, yeast cells in the culture medium were collected and washed three times by centrifugation at 16000 x *g* for 2 min at 4°C using phosphate-buffered saline (PBS pH 7.4). Yeast cells were visualised under fluorescence microscope (Olympus) at 492 nm and photographed. Following fluorescence detection, cells were analysed using flow cytometer (FACS Calibur, Becton Dickinson). A total of 30 000 yeast cells were analysed for each sample and the data analysed using FlowJo.

### Cleavage and identification of the displayed mCherry

Cells (1 ml) of Yl-mch1 and Yl-p410 cultivated for 96 h were harvested and washed three times by centrifugation at 16000 x *g* for 2 min with enterokinase buffer (20 mM Tris–HCl pH 8.0, 2 mM CaCl_2_, 50 mM NaCl_2_). Washed cells were resuspended into 1 ml of enterokinase buffer and 4 ng/ml of enterokinase (New England Biolabs, USA) was added to the cell suspension. The mixture was incubated at 16°C for 24 hours and 200 μl of the supernatant precipitated with acetone. Sodium dodecyl sulphate polyacrylamide gel electrophoresis (SDS-PAGE) was performed in a 12% polyacrylamide gel under denaturing conditions ([Laemmli 1970]).

Protein band at approximately 25 kDa on SDS gel was excised and cut into small chips. The sample was treated as described by [Webster and Oxley (2005]) and digested overnight with porcine trypsin (Promega, Madison, USA). MALDI-TOF-MS was performed using a QSTAR® Elite mass spectrometer (Applied Biosystems Inc., Ontario, Canada). The generated PMF data was searched against SWISS-PROT/TrEMBL release 35, using Protein Probe (Micromass), or against a non-redundant database maintained by the National Center for Biotechnology Information (NCBI) using the Mascot (Matrix Science Inc., Boston, MA, USA) search engine ([Helsens et al. 2007]).

## Results

### Immobilization of mCherry protein on *Y. Lipolytica*

**Figure 2:**
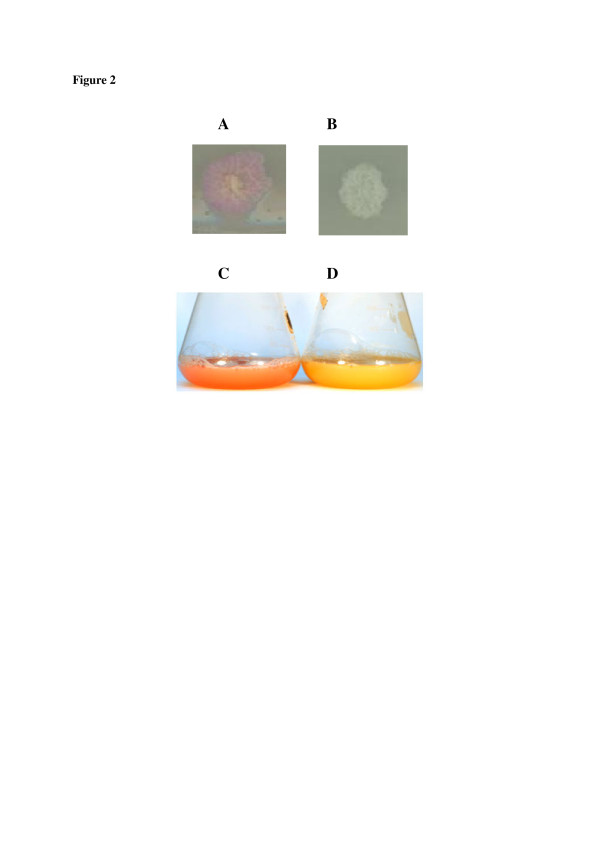
**Effect of displayed mCherry on the*Y. lipolytica*transformants and culture media.** ( **A**) Photograph of cells carrying mCherry-YlCWP1, the cells showed a pinkish-reddish colour. ( **B**) Photograph of the control cells carrying YlCWP1, cells did not show any colour change. ( **C**) Photograph of shake flask culture media containing *Y. lipolytica* displaying mCherry proteins, the culture medium showed a change in colour during the cultivation period which was a result of the displayed mCherry. ( **D**) Photograph of the shake flask culture media containing *Y. lipolytica* control cells, culture media did not show any change in colour during the cultivation period.

To evaluate the rDNA based vector using YlCWP1 GPI-anchored protein for cell surface display on *Y. lipolytica* cell wall, a multi-copy plasmid for the display of mCherry as a model protein was constructed (pKOV410- *mcherry-YlCWP1*, Figure 1a). For displaying mCherry on the cell surface of *Y. lipolytica*, its encoding sequence containing an enterokinase cleavage site at its C-terminal was fused to the N-terminal of the *YlCWP1* encoding sequence. The *mcherry* and *YlCWP1* fusion gene was linked by an enterokinase cleavage site (DDDDK) which is essential for cleavage of mCherry protein from the yeast cell surface after expression. The fusion gene was inserted into the multi-cloning site of the multi-copy plasmid, pKOV410, downstream of the pre-pro Lip2 secretion signal under the control of hp4d promoter. The expression cassettes were transformed into *Y. lipolytica* Po1f using the method of ([Xuan et al. 1988]). *Y. lipolytica* transformants carrying *mCherry* were confirmed by PCR (data not shown). Yl-mch1 transformants growing on YNB-casamino selective plates showed a purple colour (Figure 2A) compared to Yl-p410 control transformants (Figure 2B). The change in colour of Yl-mch1 transformants indicated that mCherry was displayed on the *Y. lipolytica* cells. Yl-mch1 transformed cells exhibited reddish colour even when grown in YPD liquid medium (Figure 2C) in comparison to the control cells (Figure 2D).

**Figure 3:**
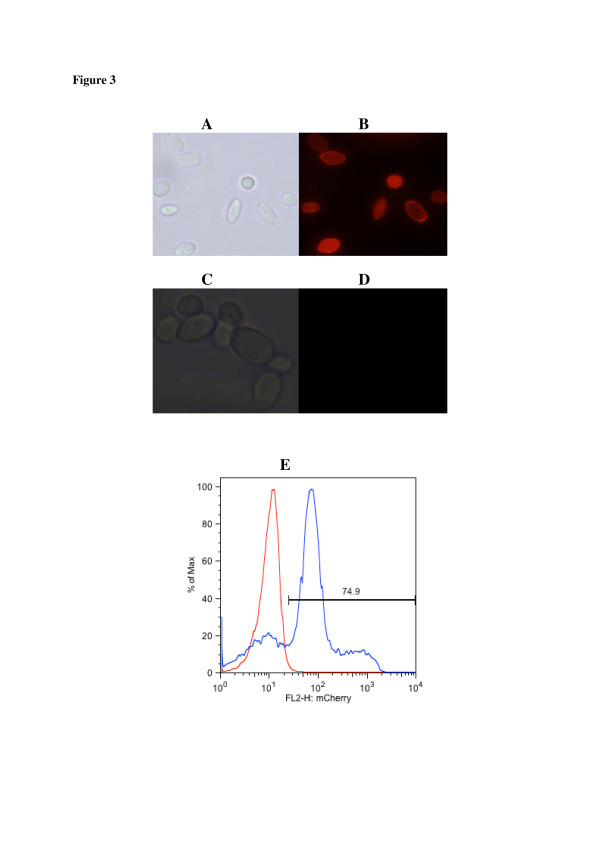
**Microscopic and flow cytometric photographs of*Y. lipolytica*cells transformed with pKOV410-*mCherry-YlCWP1*(A & B) and pKOV410-*YlCWP1*(C & D) expression cassettes.***Y. lipolytica* cells in A and C were photographed under visible light and the cells in B and D were photographed under UV light (492 nm). Flow cytometry histograms shown in ( **E**) depict the mean fluorescent signal of mCherry displayed on *Y. lipolytica* cells (in blue) and control cells (in red).

Successful display of mCherry on the cell surface of cultivated *Y. lipolytica* transformants was also confirmed by fluorescent microscopy. Yl-p410 cells were used as control. Fluorescence results in Figure 3 showed a strong fluorescence indicating that Yl-mch1 cells successfully displayed mCherry (Figure 3B), whereas no fluorescence was observed on the control cells (Figure 3D). Quantitative expression of mCherry on Yl-mch1 cells was analysed using flow cytometry (Figure 3E). About 75% of Yl-mch1 cells expressed mCherry on their surface. These results strongly suggest that mCherry was successfully expressed on the cell surface of *Y. lipolytica*.

### Cleavage of mCherry displayed on *Y. Lipolytica* cells

**Figure 4:**
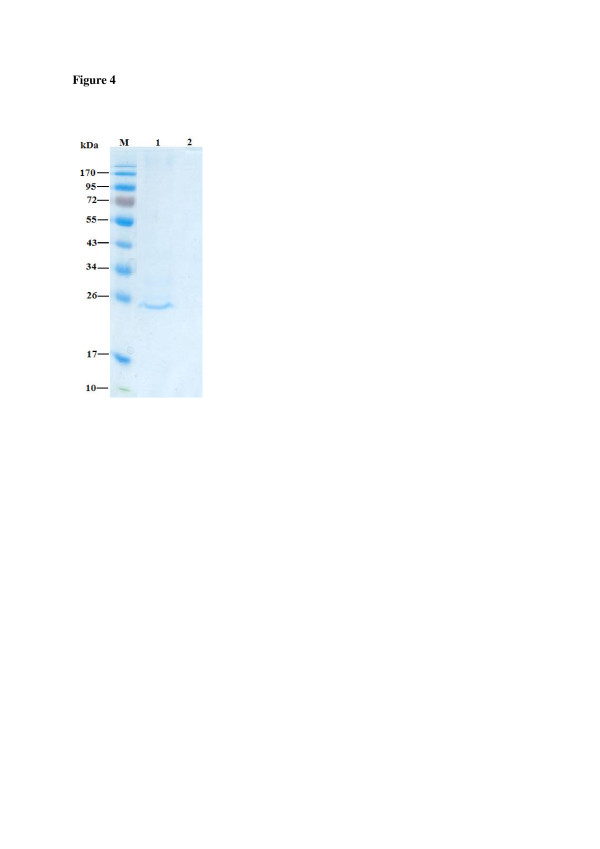
**SDS-PAGE analysis of mCherry cleaved from*Y. lipolytica*cell surface.** Lane M: Prestained protein molecular weight marker (NEB); Lane 1: m-Cherry cleaved from *Y. lipolytica* cells with enterokinase; Lane 3: *Y. lipolytica* control cells treated with enterokinase.

In order to obtain free mCherry, Yl-mch1 cells were treated with enterokinase. An approximately 25 kDa protein was detected on SDS-PAGE (Figure 4). To ascertain that the observed protein band on the SDS-PAGE was mCherry, the band was subjected to MALTI-TOF-MS peptide mass fingerprinting. The generated data was analysed using Mascot BLAST and NCBI. BLAST searches of the 23 identified peptide mass fingerprints gave 95% match to a synthetic monomeric red fluorescent protein (Gen Bank Accession number: **AAV52164**). These results indicate that mCherry was successfully displayed on *Y. lipolytica* cells. Additionally, the displayed mCherry could be removed from the cell surface by treatment with enterokinase.

## Discussion

Cell surface display has shown great potential for various applications, such as whole-cell biocatalysis and combinatorial library construction [(Furukawa et al. 2006]). Its application in *Y. lipolytica*, which has been reported to secrete a wide range of proteins ([Beckerich et al. 1998]), could be essential for immobilization of active heterologous proteins. In this study, we constructed a novel rDNA based plasmid for surface display of heterologous proteins on *Y. lipolytica*. The plasmid of [Yue et al. (2007]) and ([Yang et al. 2009]) employ *ura3d1* and *LEU2* selection markers for single copy integration into the zeta targeting sequence and the pBR322 docking platform, respectively [(Madzak et al. 2000]; [Nicaud et al. 2002]). In addition, vectors carrying both a zeta sequence and *ura3d4* are integrated into *Y. lipolytica* genome with low transformation frequencies [(Juretzek et al. 2001]). The plasmid developed in this study uses *ura3d4* allele for homologous multiple integration into the rDNA cluster [(Juretzek et al. 2001]). Similar to the zeta-based plasmid constructed by ([Yue et al. 2007]) for cell surface display of heterologous proteins on *Y. lipolytica*, the constructed rDNA based displaying plasmid in this study uses a strong recombinant growth phase dependent hp4d promoter [(Madzak et al. 2004]). The hp4d promoter has traits optimal for heterologous protein expression as it operates almost unaffected by environmental conditions such as pH, carbon and nitrogen sources and presence of peptones [(Madzak et al. 1995,^2000^]). In addition, the expression cassette used to transform *Y. lipolytica* is devoid of a bacterial moiety including antibiotic resistance genes as a result retaining its GRAS (generally regarded as safe) status ([Nicaud et al. 1998]; [Pignede et al. 2000a]). As a result of these characteristics, the plasmid pKOV410 was used to construct a novel plasmid for enhanced cell surface display on *Y. lipolytica* using the GPI-anchored *YlCWP1*. The efficiency of the displaying plasmid for enhanced display of heterologous proteins was demonstrated using mCherry as a model protein. When *Y. lipolytica* was transformed with the expression cassettes, purple transformants were observed on the YNB-casamino selective plates compared to the *Y. lipolytica* negative control transformants (Figure 2). This colour change on the transformants served as a quick visual indication that mCherry was displayed on the yeast cell surface. The purple colour was observed on cells grown on both solid agar plates and liquid YPD medium, respectively (Figure 2A & C). Similar results were first reported by ([Keppler-Ross et al. 2008]) in *S. cerevisiae* overexpressing codon optimised enhanced monomeric red fluorescent protein (EmRFP). In addition, [(Gerami-Nejad et al. 2009]) constructed a synthetic codon optimised monomeric red fluorescent protein from *Discosoma sp.* (DsRed) that produced transformants detectable at colony level based on colour. Recent studies by [(Wu et al. 2011]) demonstrated a change in colour of *E. coli* cells displaying enhanced green fluorescent protein (EGFP) after induction with IPTG. However, the fluorescence was insufficient to change the colour of the growth medium to green. Studies by [(Kuroda et al. 2009]) did not report transformants colour changes when the DsRed-monomer was displayed on *S. cerevisiae* cell surface. To our knowledge, the purple colour change observation in the Yl-mch1 engineered in this study constitutes the first report of such an effect of displayed mCherry on both solid and liquid medium.

Yl-mch1 cells exhibited red fluorescence under fluorescent microscopy, but no red fluorescence was observed on the control cells (Figure 3). The fluorescent protein, mCherry, emits in the red wavelengths of the visible spectrum [(Castro-Longoria et al. 2010]). Previous studies using the GPI-anchored YlCWP1 have reported successful display of heterologous proteins on *Y. lipolytica* cell surface [(Yue et al. 2007]; [Ni et al. 2009]; [Liu et al. 2009,^2010^]; [Yu et al. 2010]). The expression level of displayed mCherry on *Y. lipolytica* cell wall was evaluated using flow cytometry (Figure 3)E. The results indicate that approximately 75% of Yl-mch1 cells displayed mCherry. Following treatment of the cells with enterokinase, free mCherry was detected on SDS-PAGE (Figure 4) and confirmed by MALDI-TOF-MS peptide mass fingerprinting. Most studies have been unable to report detection of free heterologous protein on SDS-PAGE after cleavage of the displayed proteins on cells. Previous studies have relied on western blot analysis for detection of cleaved proteins [(Wang et al. 2007,^2008^]; [Jiang et al. 2007]). Because of enhanced expression of mCherry on the cell surface of *Y. lipolytica*, high levels of mCherry were observed on the yeast cells after cleavage with enterokinase as indicated by the purple colour phenotype. This incomplete cleavage of mCherry from the cell surface is probably due to inefficient proteolysis and could require optimization for complete cleavage. Accessibility of displayed heterologous proteins on yeast cells for cleavage has been investigated by ([Kuroda et al. 2009]) using DsRed-monomer as a model on *S. cerevisiae* cells. More recently, ([Wu et al. 2011]) reported detection of free recombinant EGFP on SDS-PAGE. Unlike the studies by ([Wu et al. 2011]) which purified EGFP using the histidine tag, the mCherry in this study did not require any purification as it was cleaved as a single dominant protein.

In this study, we have developed a method for high level expression of mCherry on *Y. lipolytica* cell surface using an rDNA based cell surface displaying plasmid. As a result of colour development and visual detection of the transformed colonies, the rDNA based plasmid together with mCherry could be used for visual screening in identifying new cell wall proteins in *Y. lipolytica*. This method will be similar to that used for selection of white and blue bacterial colonies associated with the disruption of *lacZ*-encoded β-galactosidase and those reported by ([Keppler-Ross et al. 2008]) and ([Gerami-Nejad et al. 2009]). The constructed plasmid offers an alternative approach for the purification of heterologous proteins displayed on *Y. lipolytica* cell wall. Displayed proteins can be cleaved with enterokinase to obtain pure proteins without the need to purify using chromatographic methods. The developed system is highly efficient for downstream processing of displayed heterologous proteins. Cell surface display has great potential for various applications such as whole-cell biocatalysis and combinatorial library construction ([Furukawa et al. 2006]). Application of cell surface display on *Y. lipolytica* could be essential for immobilization and purification of displayed heterologous proteins at lower costs. The developed displaying system offers great potential for industrial expression and purification of heterologous proteins.

## **Competing interests**

The authors declare that they have no competing interests.
